# Multiple-Locus Variable-Number Tandem-Repeat Analysis of *Mycoplasma pneumoniae* Clinical Specimens and Proposal for Amendment of MLVA Nomenclature

**DOI:** 10.1371/journal.pone.0064607

**Published:** 2013-05-30

**Authors:** Hongmei Sun, Guanhua Xue, Chao Yan, Shaoli Li, Ling Cao, Yi Yuan, Hanqing Zhao, Yanling Feng, Liqiong Wang, Zhaoyang Fan

**Affiliations:** 1 Department of Bacteriology, Capital Institute of Pediatrics, Chaoyang District, Beijing, China; 2 The Affiliated Children's Hospital of Capital Institute of Pediatrics, Chaoyang District, Beijing, China; 3 Department of Integrated Early Childhood Development, Capital Institute of Pediatrics, Chaoyang District, Beijing, China; Miami University, United States of America

## Abstract

*Mycoplasma pneumoniae* is one of the major respiratory bacterial pathogens that cause pneumonia in humans. Multiple-locus variable-number tandem-repeat analysis (MLVA) is currently the most discriminative method for typing *M. pneumoniae* strains. To better understand the epidemic of *M. pneumoniae*-related pneumonia in pediatric patients in Beijing, China, we performed MLVA analysis on 118 specimens collected during an epidemic from 2010–2012. Eleven distinct MLVA types were identified, including four novel types. There was no obvious association of macrolide resistance with any of the genotypes. Considering the instability of VNTR locus Mpn1, we propose an amended MLVA nomenclature system based on the remaining four VNTR loci.

## Introduction

*Mycoplasma pneumoniae* is a common respiratory bacterial pathogen that causes pneumonia in humans, especially in children and young adults [1], [2]. The infection is transmitted through close contact with an infected patient, and leads to epidemics in the immediate family and community. Worldwide epidemics of *M. pneumoniae* infection occur every 3 to 7 years [3], [4]. Studies show that an epidemic has been spreading in many countries since 2010 [5], [6], [7], [8], [9], [10]. To study an epidemic and identify the source of an outbreak, it is important to type the pathogenic strains rapidly and accurately.

Molecular typing of the *M. pneumoniae* P1 gene has been the most common genotyping method in the past 30 years. Two major types of P1 genes (types 1 and 2) [11], [12], [13], three subtypes, and three variants of these subtypes have been described [14], [15], [16], [17], [18]. Recently, a multiple-locus variable-number tandem-repeat (VNTR) analysis (MLVA) for molecular typing of *M. pneumoniae* was developed [19]. Twenty-six MLVA types (A–Z) were first introduced by typing 265 *M. pneumoniae* strains using five VNTR loci (Mpn1, Mpn13–16). This highly-discriminatory method was quickly adapted by other research groups, and at least 18 novel types have been reported [20], [5], [6], [21], [22], [8], [23]. However, locus Mpn1 is unstable in both clinical strains and in laboratory passages, and most of the novel types came from variations in Mpn1 [20], [8], [23]. With more MLVA genotypes identified, the current *M. pneumoniae* MLVA nomenclature system needs to be amended.

In this study, we typed 118 clinical specimens collected from pediatric patients in Beijing, from 2010–2012, by MLVA and P1-RFLP. We also analyzed all reported MLVA types in the literature and propose a modified nomenclature for the *M. pneumoniae* MLVA types.

## Materials and Methods

### Ethics Statement

The present project was performed in compliance with the Helsinki Declaration (Ethical Principles for Medical Research Involving Human Subjects) and was approved by the research board of the Ethics Committee of the Capital Institute of Pediatrics, Beijing, China. All patient data were anonymously reported. Based on the guidelines of the Ethics Committee of the Capital Institute of Pediatrics this study did not need to be examined by the ethical committee, and therefore informed consent was not sought from patients.

### Clinical Specimens, DNA Extraction, and Real-time PCR Detection

A collection of 681 clinical specimens was obtained from pediatric patients diagnosed with pneumonia or with respiratory tract infections in the Affiliated Children's Hospital of the Capital Institute of Pediatrics in Beijing. The specimens consisted of sputum samples, throat swabs, bronchoalveolar lavage fluid, and puncture fluid, of which 178 specimens were collected in 2010, 159 in 2011, and 344 in 2012. Each specimen was collected from a single patient. DNA samples were extracted using the TIANcombi DNA Lyse & Amp PCR Kit (Tiangen, Hangzhou, China) according to the manufacturer’s instructions. Briefly, 0.4 mL of the specimen was centrifuged at 13,800×*g* for 15 min and the pellet was resuspended in 0.5 mL of lysis buffer (150 mM NaCl, 20 mM sodium citrate, 10 mM Tris-HCl, 1 mM EDTA, 1 mg/mL proteinase K, 20 mg/mL RNase A, 1% (w/v) sodium dodecyl sulfate). Following incubation at 55°C for 60 min and 100°C for 10 min, lysates were centrifuged at 13,800×*g* for 3 min. The supernatants were transferred to a purification column and the DNA was eluted in a volume of 50 µL. The DNA was immediately used for real-time PCR detection of *M. pneumoniae* [15] or stored at –20°C.

### P1 Gene Typing

To improve assay sensitivity, a nested P1 gene PCR-RFLP method was performed directly on the *M. pneumoniae*-positive samples,. To detect the RepMP4 region of the P1gene, the ADH1/ADH2 primer pair [13] was used for the first PCR, and the ADH1in/ADH1M and ADH2in/ADH2M primer pairs were designed and used for the second PCR (Table 1). To detect the RepMp2/3 region, the ADH3/ADH4 primer pair [13] was used for the first PCR, and the ADH3in/ADH3M and ADH4in/ADH4M primer pairs were designed and used for the second PCR (Table 1). The nested PCR conditions were as follow: 94°C for 10 min, 30 cycles of 94°C for 1 min, 55°C for 1 min, and 72°C for 2 min, followed 72°C for 10 min. PCRs were performed on a Thermal cycle PX2 apparatus (Becton Dickinson, Franklin Lakes, NJ). The nested PCR products of the RepMP4 and RepMp2/3 regions were digested using the restriction enzyme *Hae*III and were run on a 2% agarose gel. The restriction fragment length polymorphism (RFLP) was analyzed as described [13].

**Table 1:** Primers used in P1-RFLP gene typing.

Primer	Sequence *(5′–3′)	Tm (°C)
ADH1in	180900 ATTCTCATCCTCACCGCCA 180918	65.68
ADH1M	182049 CCTTGGGATCCAAGTGATCA 182030	64.84
ADH2in	183090 CTGCTAACAATTCCGGATTGAG 183069	64.43
ADH2M	182030 TGATCACTTGGATCCCAAGG 182049	64.84
ADH3in	183130 TTGCTGCTAACGAGTACGAG 183149	60.76
ADH3M	184394 CAAATCCCACACACTCTCCA 184375	63.65
ADH4in	185623 TGACTGATACCTGTGCGG 185606	60.92
ADH4M	184375 TGGAGAGTGTGTGGGATTTG 184394	63.65

* Reference strain: U00089 (M.

### MLVA Typing

MLVA was performed based on a previously described method, with modifications [19]. First, a nested PCR that had been adapted for direct clinical specimens was carried out using outer and inner primers targeting the five selected loci containing tandem repeats (TRs, Mpn1, Mpn13, Mpn14, Mpn15, Mpn16) [21]. Briefly, a 25 µL reaction mixture containing 3 µL extracted DNA, 0.5 µL each of forward and reverse primer (10 µmol/L), and 12.5 µL 2× Qiagen HotStar*Taq* polymerase mix was run under cycling conditions of 95°C for 10 min, followed by 35 cycles of 95°C for 30 s, 55°C for 30 s, and 72°C for 1 min, and a final extension at 72°C for 10 min. The PCR products were sequenced and the VNTR copy numbers were counted from the sequence results (Supporting Information).

In this study, to fully describe a strain using the molecular signatures from both typing methods, the MLVA type was designated by a prefix of M plus the number of repeats at each locus in the order of Mpn1-Mpn13-Mpn14-Mpn15-Mpn16 (Mn-n-n-n-n) [24]. The combination of the MLVA and P1 typing was presented as Mn-n-n-n-nPn (M-P).

### Detection of Macrolide Resistance Gene

The point mutations at positions 2063, 2064, 2616, and 2617 of the 23S rRNA gene of *M. pneumoniae*, which are responsible for macrolide resistance, were detected by a nested PCR-linked capillary electrophoresis and single-strand conformation polymorphism analysis (nPCR-CE-SSCP) based on a previous method [25].

The PCR products were purified and sequenced to verify mutations.

### Data Analysis

The numbers of repeats identified at the five loci in the MLVA typing were recorded and imported into the Bionumerics (version 5.0) software package (Applied Maths). A minimum-spanning tree (MST) was generated based on categorical coefficient, and the priority rule was set as the highest number of single locus variants (SLVs). The polymorphism indexes of P1 gene typing, MLVA typing, and MLVA plus P1 (M-P) typing were calculated using the Hunter-Gaston diversity index (HGDI) [26].

## Results

### Detection of *M. pneumoniae* from the Clinical Specimens

**Figure 1:**
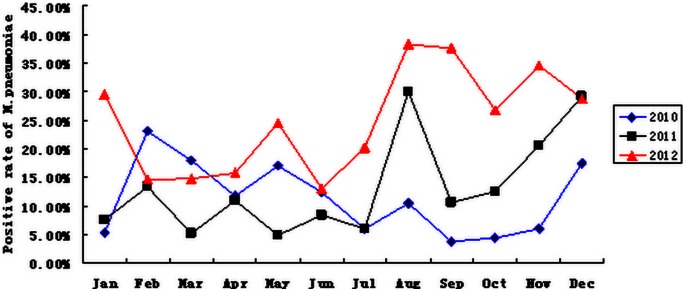
Positive rate of *M. pneumoniae* detection from January 2010–December 2012.

Of the 681 clinical samples, 118 specimens (17.3%) were positive for *M. pneumoniae* by real-time PCR. Of the positive samples, 20 (11.2%, 20/178) were collected in 2010, 21 were from 2011 (13.2%, 21/159), and 77 (22.4%, 77/344) were collected in 2012. The infection rate increased from late 2011, and was highest in August (28.8%) of 2011, and January (29.5%), August (38.3%), September (37.6%), October (26.7%), November (34.6%), and December (28.8%) of 2012 (Fig. 1). Of the 118 *M. pneumoniae* positive patients (55 boys/63 girls with an age range of 0.7–13.2 years), 92, 20, 2, and 4 were diagnosed with pneumonia, bronchopneumonia, capillary bronchitis, and respiratory tract infections, respectively (Table 2). The average macrolide treatment and hospitalization periods were 16 days and 15.6 days, respectively.

**Table 2:** Characteristics of the 116 *M. pneumoniae* specimens used in this study for MLVA and P1 typing.

M-P type	No. of samples	No. of A2063G specimens/No. of tested specimens	Mpn 1-13-14-15-16	P1 typing	Typing by Dégrange	Sex (M/F)	Age (year)	Diagnosis	Total days of using macrolide	Average hospitalization days
M2-4-5-7-2P1	10	4/4	2-4-5-7-2	1	E	5/5	2.6–12.1	Bronchopneumonia, pneumonia	17	16.6
M2-4-5-7-3P1	1		2-4-5-7-3	1	*	1/0	5.0	pneumonia	14	13
M3-3-5-6-2P2	1	1/1	3-3-5-6-2	2	G	0/1	2.6	Bronchopneumonia	19	17
M3-4-5-7-2P1	15	8/8	3-4-5-7-2	1	J	5/10	1.25–12.1	Bronchopneumonia, pneumonia pleural effusion	14.6	12.8
M4-4-5-6-2P1	2	1/2	4-4-5-6-2	1	*	0/2	0.8–2.7	Respiratory tract infection, pneumonia	15	13
M4-4-5-7-2P1	25	11/12	4-4-5-7-2	1	P	14/11	0.7–12.1	severe pneumonia, pneumonia, bronchopneumonia	17.2	16.9
M5-4-5-7-2P1	31	15/17	5-4-5-7-2	1	U	13/18	1.7–10.8	bronchopneumonia, pneumonia, pleural effusion,severe pneumonia, pulmonary atelectasis	19.4	16.6
M5-4-5-7-2P2	1	1/1	5-4-5-7-2	2	U	1/0	5.8	bronchopneumonia	23	16
M6-4-5-6-2P1	1	1/1	6-4-5-6-2	1	*	0/1	7.6	pneumonia	20	23
M6-4-5-7-2P1	21	12/14	6-4-5-7-2	1	X	14/7	2.1–13.2	respiratory tract infection, bronchopneumonia, pneumonia	10	14.6
M7-4-5-7-2P1	5	2/2	7-4-5-7-2	1	Z	0/5	1.8–10.0	pneumonia, pulmonary atelectasis, bronchopneumonia,	25	19.5
M8-4-5-7-2P1	3	3/3	8-4-5-7-2	1	*	2/1	3.3–9.3	respiratory tract infection, pneumonia	12	9

Samples could not be classified according to the published scheme based on the 26 combinations of the number of tandem repeats assigned to the alphabetic characters A to Z.

### P1 Gene Typing

PCR-RFLP analysis of the P1 gene showed that, among the 118 PCR positive samples, 116 (98.3%) were P1 type 1, while the remaining two (1.7%) were P1 type 2.

### MLVA Typing

Eleven distinct MLVA types were identified from 116 out of the 118 samples (Table 2). It was not possible to obtain the full MLVA profile from two positive samples, which may be due to the low levels of DNA in the samples. Of the 11 genotypes identified, seven were reported previously by Dégrange [19], including E (n = 10), J (n = 15), P (n = 25), U (n = 32), X (n = 21), Z (n = 5) and G (n = 1), and four types were novel profiles M2-4-5-7-3 (n = 1), M4-4-5-6-2 (n = 2), M6-4-5-6-2 (n = 1), and M8-4-5-7-2 (n = 3). The most prevalent genotypes were U (27.6%), P (21.6%), X (18.5%), and J (12.9%).

Because the number of the patients with each of the genotypes was small, the associations between the genotype and sex, age, the average days of using macrolide and hospitalization, and the clinical severity of the patients could not be analyzed.

The discriminatory power of the MLVA was 0.821, compared with 0.838 for MLVA combined with P1 typing, and 0.05 for P1 typing alone. Combining the P1 typing result with MLVA showed there were 12 distinct M-P types from the 116 clinical specimens, with a separation of two U types: M5-4-5-7-2P1 (U) and M5-4-5-7-2P2 (U) (Table 2).

Examining the originally reported 26 MLVA types, we found that locus Mpn1 is highly variable, while the other four loci are more stable. If locus Mpn1 is omitted and only the other four loci are considered, the 26 MLVA types (A–Z) become nine types (Table 3). Using this simplified system, the MLVA types reported in the literature could be classified into 15 genotypes from type 2-6-6-2 to 4-6-7-2 (Table 4) [1,2, 3, 4, 6, 8,18, 19, 36]. The majority of the samples from this study (111/116, 95.7%) were type 4-5-7-2 (Table 4). This might indicate an association of a certain strain with this epidemic in pediatric patients in Beijing.

**Table 3:** VNTRs (Mpn13, 14, 15, 16) in 26 genotypes (A–Z).

MLVA typing by Dégrange	Number of repeats in Mpn1	Number of repeats in Mpn13-14-15-16
L	4	2-6-6-2
B/G/M/S/V/Y	2/3/4/5/6/7	3-5-6-2
N/R	4/5	3-5-7-2
C/H/O/T/W	2/3/4/5/6	3-6-6-2
I	3	3-6-7-2
D/K	2/3	4-5-6-2
Q	4	4-5-7-1
A/E/J/P/U/X/Z	1/2/3/4/5/6/7	4-5-7-2
F	2	4-5-8-2

**Table 4:** Fifteen MLVA types divided according to the repeat numbers of Mpn13, 14, 15, 16.

No. Of type	4-locus MLVA type	MLVA	Mpn1	Mpn13-14- 15-16	Reported by (year of report)
1	2-6-6-2	L	4	2-6-6-2	Dégrange(2009)
2	3-4-7-2	*	2	3-4-7-2	Dumke(2011)
3	3-5-6-2	B	2	3-5-6-2	Dégrange(2009),Dumke(2011),Chalker(2011), Pereyre(2012), Benitez(2012)
		G	3	3-5-6-2	Dégrange(2009),Dumke(2011),Pereyre(2012), Benitez(2012), Zhao2012,Sun (present study)
		M	4	3-5-6-2	Dégrange(2009),Dumke(2011),Chalker(2011,2012), Pereyre(2012), Benitez(2012), Zhao2012
		S	5	3-5-6-2	Dégrange(2009),Dumke(2011),Chalker(2012), Pereyre(2012), Benitez(2012)
		V	6	3-5-6-2	Dégrange(2009),Dumke(2011),Chalker(2011), Pereyre(2012), Benitez(2012), Zhao2012
		Y	7	3-5-6-2	Dégrange(2009),Dumke(2011)
4	3-5-7-2	N	4	3-5-7-2	Dégrange(2009)
		R	5	3-5-7-2	Dégrange(2009)
		*	7	3-5-7-2	Dumke(2011)
5	3-5-7-3	*	5	3-5-7-3	Chalker(2012)
6	3-6-6-2	C	2	3-6-6-2	Dégrange(2009),Chalker(2011),Pereyre(2012),Benitez(2012)
		H	3	3-6-6-2	Dégrange(2009),Dumke(2011),Pereyre(2012), Benitez(2012)
		O	4	3-6-6-2	Dégrange(2009),Dumke(2011),Pereyre(2012), Benitez(2012)
		T	5	3-6-6-2	Dégrange(2009),Dumke(2011),Chalker(2012), Pereyre(2012), Benitez(2012)
		W	6	3-6-6-2	Dégrange(2009),Dumke(2011),Pereyre(2012), Benitez(2012)
7	3-6-7-2	I	3	3-6-7-2	Dégrange(2009),Pereyre(2012)
8	4-4-7-2	*	4	4-4-7-2	Zhao2012
		*	5	4-4-7-2	Zhao2012
		31	8	4-4-7-2	Pereyre(2012)
9	4-5-6-2	D	2	4-5-6-2	Dégrange(2009)
		K	3	4-5-6-2	Dégrange(2009)
		*	4	4-5-6-2	Sun (present study)
		*	6	4-5-6-2	Dumke(2011),Sun (present study)
10	4-5-7-1	Q	4	4-5-7-1	Dégrange(2009)
11	4-5-7-2	A	1	4-5-7-2	Dégrange(2009),Pereyre(2012)
		E	2	4-5-7-2	Dégrange(2009),Dumke(2011),Chalker(2011,2012), Pereyre(2012), Benitez(2012),Zhao2012,Sun (present study)
		J	3	4-5-7-2	Dégrange(2009),Dumke(2011),Chalker(2011), Pereyre(2012), Benitez(2012), Zhao2012,Sun (present study)
		P	4	4-5-7-2	Dégrange(2009),Dumke(2011),Chalker(2011,2012), Pereyre(2012), Benitez(2012), Zhao2012,Sun (present study)
		U	5	4-5-7-2	Dégrange(2009),Dumke(2011),Chalker(2011,2012), Pereyre(2012), Benitez(2012), Zhao2012,Sun (present study)
		X	6	4-5-7-2	Dégrange(2009),Dumke(2011),Pereyre(2012), Benitez(2012), Zhao2012,Sun (present study)
		Z	7	4-5-7-2	Dégrange(2009),Dumke(2011),Chalker(2011,2012), Pereyre(2012), Benitez(2012), Zhao2012,Sun (present study)
		*	8	4-5-7-2	Zhao2012,Sun (present study)
12	4-5-7-3	*	2	4-5-7-3	Zhao2012, Sun (present study)
		*	3	4-5-7-3	Zhao2012
		*	4	4-5-7-3	Chalker(2011), Zhao2012
		*	5	4-5-7-3	Zhao2012
		28	6	4-5-7-3	Chalker(2012)
13	4-5-8-2	F	2	4-5-8-2	Dégrange(2009)
14	4-6-6-2	30	3	4-6-6-2	Pereyre(2012)
15	4-6-7-2	29	2	4-6-7-2	Dumke(2011)
		27	3	4-6-7-2	Chalker(2011)
		*	4	4-6-7-2	Benitez(2012)
		*	5	4-6-7-2	Benitez(2012)

* Genotype with the new number combinations that could not be identified by the 26-letter method.

### Detection of the Macrolide Resistance Mutation in the 23S rRNA Gene

Mutation detection by nPCR-CE-SSCP was successfully carried out in 65 *M. pneumoniae* positive specimens. Fifty-nine specimens (90.8%) had a characteristic mutation of A>G at position 2063 by nPCR-CE-SSCP, which was confirmed by sequencing (Table 2). This mutant genotype was distributed in all eleven distinct M-P types. Among the other six wild-type specimens, two were grouped into genotype M5-4-5-7-2P1 (U, 2/17, 11.8%), two were M6-4-5-7-2P1 (X, 2/14, 14.3%), and the other two were M4-4-5-7-2P1 (P, 1/12, 8.3%) and M4-4-5-6-2P1 (1/2, 50.0%), respectively.

## Discussion

The most recent epidemic outbreak of *M. pneumoniae* infection was reported in Denmark in late 2010 [10], and then a similar increase in *M. pneumoniae* infection was also noted in England and Wales over the same period [5]. In 2011, *M. pneumoniae* infection was reported in many other European and Asian countries [27, 28, 29, 3, 7, 9 and 30]. This study suggests that an epidemic outbreak also occurred in Beijing, China, during this period.

P1 gene PCR-RFLP has been the most common method for molecular typing of *M. pneumoniae*. The method only divided the strains into two types: types 1 and 2, while type 2 strains could be further subtyped into 2a, 2b, and 2c [31], [14], [32], [8], [33]. A type shift phenomenon was reported during different epidemic periods: type 2 strains were predominant between 1995 and 2001 [32], while type 1 strains were prevalent between 2002 and 2005 [32]. This study indicated that type 1 was the predominant genotype in the Beijing epidemic between 2010 and 2012. This was consistent with reports from China and France in 2011 [18], [34].

The discriminatory power of MLVA is much higher than the P1 typing method. Combining MLVA typing with P1 typing provided a limited increase in discriminatory power, as shown in this study. While P1 typing is the gold standard of *M. pneumoniae* typing and has been used for over 30 years, it might be useful to compare the current results with the historical data. However, an open discussion of whether to continue using P1 typing in the future is encouraged.

**Figure 2:**
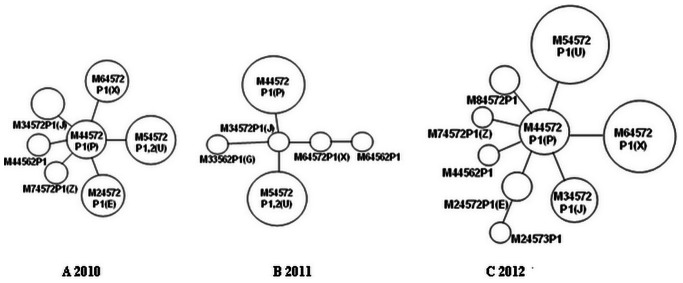
Minimum spanning trees for *Mycoplasma pneumoniae* detected in Beijing, 2010 (n = 20, A), 2011 (n = 21, B), and 2012 (n = 75, C). Each circle represents a unique genotype. The size of each circle illustrates the proportion of specimens with that genotype. The smallest circle in each tree represents one specimen. Two adjacent circle connected by a line had a single locus variant.

In this study, genotypes P (M4-4-5-7-2P1), U (M5-4-5-7-2P1), X (M6-4-5-7-2P1), and J (M3-4-5-7-2P1) were predominant between 2010 and 2012 in Beijing based on the MST analysis (Fig. 2). Because the first VNTR locus, Mpn1, is located in a hypervariable region of the genome, it may not meet the VNTR selection standard [20, 27, 5, 7 and 8]. If Mpn1 was removed from the typing scheme, the prevalent genotype in Beijing during the *M. pneumoniae* epidemic between 2011 and 2012 becomes 4-5-7-2 (P, U, X, and J). Using the same approach to analyze a recent report, also from Beijing from 2008–2011, the most common type were also found to be 4-5-7-2 (E, J, P, U, X, and Z, 174/201), and other two types 3-5-6-2 (G, M, and V, 15/201) and 4-5-7-3 (10/201) were also found in this study [23]. In addition, the most prevalent genotype in Europe was type 4-5-7-2 (E, J, P, U and Z), followed by type 3-5-6-2 (B, M and V), and type 3-6-6-2 (T) in 2011/12 [27], [5], [6], [19], [21], [8]. This is very similar to the result obtained in Israel in 2010, where types 3-6-6-2 (C, H, O, T, and W) and 4-5-7-2 (A, E, J, P, U, X, and Z) were predominant [5], [8]. These data clearly suggested that the genotypes 4-5-7-2, 3-5-6-2, and 3-6-6-2 were circulating worldwide during this epidemic. The four VNTR loci (Mpn13-14-15-16) typing system produced a clearer and simpler MLVA result. We thus propose this amended four-locus *M. pneumoniae* MLVA naming system for future use. In addition, the highly variable Mpn1 locus could serve as a strain-tracking tool in some patients.

Of particular concern is the uniformity of the definition of every tandem repeat number. As most reports used capillary electrophoresis (CE) and then GeneScan to count the number of TRs, and few reports used sequencing, there may be some difference of the TRs number between the two methods. Take M129 as an example, the genotype inferred from the CE result is 4-4-5-7-2, while the gene sequencing result gives a genotype of 4-3.2-5-6.2-2, or possibly 4-3-5-6-2. These discrepancies must be resolved for better comparison of the result of MLVA.

In this study, 90.8% specimens were shown to have macrolide resistance mutation in the 23S rRNA gene and this resistance was found to be scattered in each genotype, no association between any MLVA type and macrolide resistance was identified. This was consistent with a study from Shanghai, China [22], but differed from the report from France that found MLVA type Z was associated with resistance [8]. The macrolide-resistance prevalence is higher in China compared to those found in many other countries, a phenomenon that may be associated to the excessive use of antibiotics.

In summary, we reported an epidemic of *M. pneumoniae* in Beijing, China, during 2011–2012, with a predominant strain harboring MLVA type 4-5-7-2. An amended four-locus MLVA typing scheme was proposed for future *M. pneumoniae* molecular typing.

## Supporting Information

Figure S1 **Different VNTR copy numbers of Mpn1 counted from the sequence results.**
(TIF)
