# Supplementary figures and images for: Multiple-Locus Variable-Number Tandem-Repeat Analysis of Mycoplasma pneumoniae Clinical Specimens and Proposal for Amendment of MLVA Nomenclature
Source: PLoS One. 2013 May 30;8(5):e64607. doi: 10.1371/journal.pone.0064607 (PMC3667773; doi:10.1371/journal.pone.0064607)

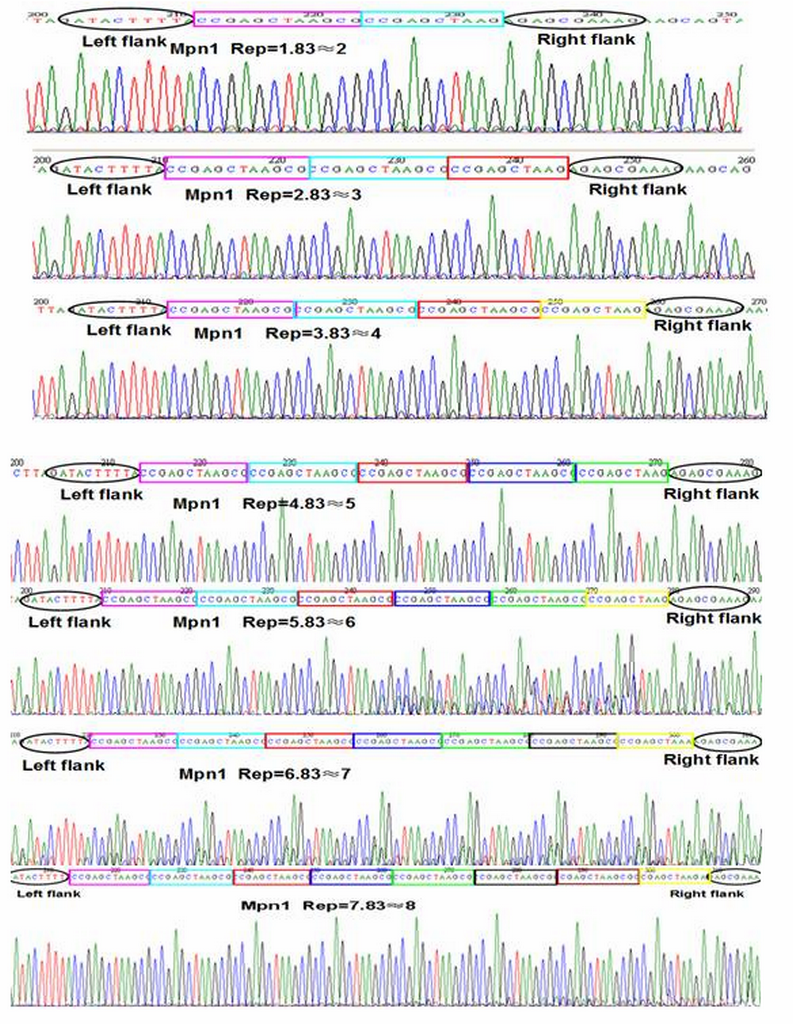

Supplement: Figure S1 — Different VNTR copy numbers of Mpn1 counted from the sequence results. (TIF) [file pone.0064607.s001.tif]
